# Poly[(μ_6_-benzene-1,3,5-tricarboxyl­ato-κ^6^ *O* ^1^:*O* ^1′^:*O* ^3^:*O* ^3′^:*O* ^5^:*O* ^5′^)tris­(*N*,*N*-dimethyl­formamide-κ*O*)tris­(μ_3_-formato-κ^2^ *O*:*O*′)trimagnesium(II)]

**DOI:** 10.1107/S1600536810035907

**Published:** 2010-09-25

**Authors:** Chun-Ting Yeh, Hsin-Kuan Liu, Chia-Jing Lin, Chia-Her Lin

**Affiliations:** a Department of Chemistry, Chung-Yuan Christian University, Chung-Li 320, Taiwan

## Abstract

The title complex, [Mg_3_(CHO_2_)_3_(C_9_H_3_O_6_)(C_3_H_7_NO)_3_]_*n*_, exhib­its a two-dimensional structure parallel to (001), which is built up from the Mg^II^ atoms and bridging carboxyl­ate ligands (3 symmetry). The Mg^II^ atom is six-coordinated by one O atom from a dimethyl­formamide mol­ecule, two O atoms from two μ_6_-benzene-1,3,5-tricarboxyl­ate ligands and three O atoms from three μ_3_-formate ligands in a distorted octa­hedral geometry.

## Related literature

For general background to the synthesis and structures of coordination polymers, see: Kitagawa *et al.* (2004 ▶); Liu *et al.* (2009 ▶). For an isotypic structure, see: He *et al.* (2006 ▶).
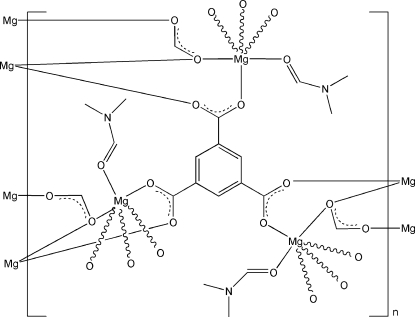


## Experimental

### 

#### Crystal data

[Mg_3_(CHO_2_)_3_(C_9_H_3_O_6_)(C_3_H_7_NO)_3_]
*M* *_r_* = 211.46
Trigonal, 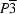

*a* = 13.9739 (2) Å
*c* = 8.1188 (1) Å
*V* = 1372.96 (3) Å^3^
*Z* = 6
Mo *K*α radiation
μ = 0.19 mm^−1^
*T* = 295 K
0.25 × 0.25 × 0.15 mm

#### Data collection

Bruker APEXII CCD diffractometer
Absorption correction: multi-scan (*SADABS*; Bruker, 2001 ▶) *T*_min_ = 0.954, *T*_max_ = 0.972
12089 measured reflections
2278 independent reflections
2212 reflections with *I* > 2σ(*I*)
*R*_int_ = 0.032

#### Refinement

*R*[*F* ^2^ > 2σ(*F* ^2^)] = 0.078
*wR*(*F* ^2^) = 0.214
*S* = 1.40
2278 reflections
129 parameters
H-atom parameters constrained
Δρ_max_ = 0.47 e Å^−3^
Δρ_min_ = −0.35 e Å^−3^

### 

Data collection: *APEX2* (Bruker, 2007 ▶); cell refinement: *SAINT* (Bruker, 2007 ▶); data reduction: *SAINT*; program(s) used to solve structure: *SHELXS97* (Sheldrick, 2008 ▶); program(s) used to refine structure: *SHELXL97* (Sheldrick, 2008 ▶); molecular graphics: *SHELXTL* (Sheldrick, 2008 ▶) and *DIAMOND* (Brandenburg, 1999 ▶); software used to prepare material for publication: *SHELXTL*.

## Supplementary Material

Crystal structure: contains datablocks I, global. DOI: 10.1107/S1600536810035907/hy2348sup1.cif

Structure factors: contains datablocks I. DOI: 10.1107/S1600536810035907/hy2348Isup2.hkl

Additional supplementary materials:  crystallographic information; 3D view; checkCIF report

## Figures and Tables

**Table 1:** Selected bond lengths (Å)

Mg1—O1	2.008 (3)
Mg1—O2	2.132 (3)
Mg1—O2^i^	2.135 (3)
Mg1—O3	2.047 (3)
Mg1—O4^ii^	2.080 (3)
Mg1—O1*S*	2.115 (4)

Symmetry codes: (i) 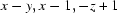; (ii) 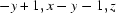.

**Fig. 1.**
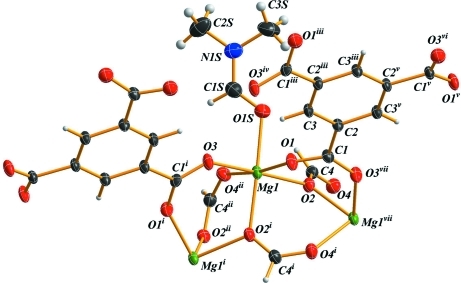
Part of the polymeric structure of the title compound, showing 50% probability displacement ellipsoids. [Symmetry codes: (i) x-y, -1+x, 1-z; (ii) 1-y, -1+x-y, z; (iii) 1-x+y, 1-x, z; (iv) 1-x, -y, 1-z; (v) 1-y, x-y, z; (vi) x-y, x, 1-z; (vii) 1+y, 1-x+y, 1-z.]

**Fig. 2.**
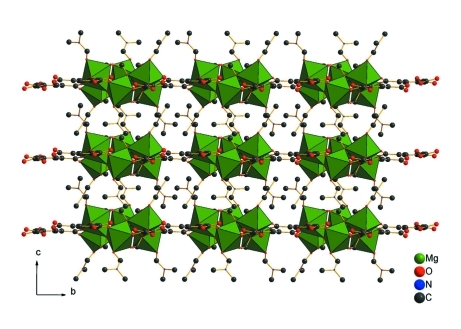
Crystal packing diagram of the title compound along the a axis.

## supplementary crystallographic information

### Comment

The synthesis of coordination polymers, or so-called metal-organic frameworks (MOFs), has been a subject of intense research owing to their interesting structural chemistry and potential applications in gas storage, separation, catalysis, magnetism, luminescence. A large number of these materials have been synthesized by solvothermal reactions with organic carboxyl acids (Kitagawa *et al.*, 2004; Liu *et al.*, 2009). They commonly adopt three-, two- or one-dimensional structures *via* employed metal ions as connectors and rigid or flexible organic ligands as linkers. As a further study of such complexes, we report the title compound (Fig. 1), which is isotypic with [Co_6_(C_9_H_3_O_6_)_2_(CHO_2_)_6_(C_3_H_7_NO)_6_]_n_ (He *et al.*, 2006) and has a two-dimensional polymeric network (Fig. 2). All the geometric parameters are within normal ranges (Table 1). The Mg^II^ atom is six-coordinated by one O atoms from an N,N-dimethylformamide molecule and five O atoms from the carboxylate ligands, giving a distorted octahedral geometry.

### Experimental

Solvothermal reactions were carried out at 423 K for 2 d in a Teflon-lined acid digestion bomb with an internal volume of 23 ml followed by slow cooling at 6 K h^-1^ to room temperature. A single-phase product consisting of transparent colorless crystals was obtained from a mixture of Mg(NO_3_)_2_.6H_2_O (0.051 g, 0.2 mmol), benzene-1,3,5-tricarboxylic acid (0.021 g, 0.1 mmol) and formic acid (0.5 ml, 0.4 *M*) and *N*,*N*-dimethylformamide (5.0 ml).

### Refinement

H atoms were constrained to ideal geometries, with C—H = 0.93 (CH) and 0.96 (CH_3_) Å and *U*_iso_(H) = 1.2(1.5 for methyl)*U*_eq_(C).

### Crystal data

**Table:**

[Mg_3_(CHO_2_)_3_(C_9_H_3_O_6_)(C_3_H_7_NO)_3_]	*D*_x_ = 1.535 Mg m^−^^3^
*M**_r_* = 211.46	Mo *K*α radiation, λ = 0.71073 Å
Trigonal, *P*3	Cell parameters from 9292 reflections
Hall symbol: -P 3	θ = 2.9–28.3°
*a* = 13.9739 (2) Å	µ = 0.19 mm^−^^1^
*c* = 8.1188 (1) Å	*T* = 295 K
*V* = 1372.96 (3) Å^3^	Columnar, colorless
*Z* = 6	0.25 × 0.25 × 0.15 mm
*F*(000) = 660	

### Data collection

**Table:**

Bruker APEXII CCD diffractometer	2278 independent reflections
Radiation source: fine-focus sealed tube	2212 reflections with *I* > 2σ(*I*)
graphite	*R*_int_ = 0.032
Detector resolution: 8.3333 pixels mm^-1^	θ_max_ = 28.3°, θ_min_ = 1.7°
φ and ω scans	*h* = −18→17
Absorption correction: multi-scan (*SADABS*; Bruker, 2001)	*k* = −17→18
*T*_min_ = 0.954, *T*_max_ = 0.972	*l* = −10→10
12089 measured reflections	

### Refinement

**Table:**

Refinement on *F*^2^	Primary atom site location: structure-invariant direct methods
Least-squares matrix: full	Secondary atom site location: difference Fourier map
*R*[*F*^2^ > 2σ(*F*^2^)] = 0.078	Hydrogen site location: inferred from neighbouring sites
*wR*(*F*^2^) = 0.214	H-atom parameters constrained
*S* = 1.40	*w* = 1/[σ^2^(*F*_o_^2^) + (0.0309*P*)^2^ + 6.185*P*] where *P* = (*F*_o_^2^ + 2*F*_c_^2^)/3
2278 reflections	(Δ/σ)_max_ < 0.001
129 parameters	Δρ_max_ = 0.47 e Å^−^^3^
0 restraints	Δρ_min_ = −0.35 e Å^−^^3^

### Fractional atomic coordinates and isotropic or equivalent isotropic displacement parameters (Å^2^)

**Table:**

	*x*	*y*	*z*	*U*_iso_*/*U*_eq_	
Mg1	0.77826 (11)	0.01439 (11)	0.39889 (18)	0.0184 (3)	
O1	0.7487 (3)	0.1240 (2)	0.5146 (4)	0.0261 (7)	
O2	0.9488 (2)	0.1369 (2)	0.3855 (4)	0.0220 (6)	
O3	0.6145 (2)	−0.1017 (2)	0.4220 (4)	0.0256 (7)	
O1S	0.7459 (3)	0.0733 (3)	0.1779 (4)	0.0327 (8)	
C2	0.7301 (3)	0.2815 (3)	0.5313 (5)	0.0181 (8)	
C3	0.6154 (3)	0.2191 (3)	0.5310 (5)	0.0188 (8)	
H3	0.5809	0.1423	0.5307	0.023*	
C4	1.0034 (3)	0.1485 (3)	0.2561 (5)	0.0223 (8)	
H4A	0.9631	0.1231	0.1590	0.027*	
C1	0.7986 (3)	0.2259 (3)	0.5408 (5)	0.0190 (8)	
N1S	0.6441 (4)	0.0408 (4)	−0.0535 (5)	0.0363 (10)	
C1S	0.6876 (4)	0.0111 (4)	0.0655 (6)	0.0328 (10)	
H1S	0.6738	−0.0612	0.0656	0.039*	
C2S	0.5833 (5)	−0.0326 (6)	−0.1892 (7)	0.0506 (16)	
H2S1	0.5853	−0.1000	−0.1790	0.076*	
H2S2	0.6165	0.0025	−0.2918	0.076*	
H2S3	0.5079	−0.0488	−0.1864	0.076*	
C3S	0.6642 (6)	0.1535 (6)	−0.0614 (8)	0.0498 (15)	
H3S1	0.6850	0.1869	0.0456	0.075*	
H3S2	0.5982	0.1525	−0.0968	0.075*	
H3S3	0.7227	0.1954	−0.1383	0.075*	
O4	1.1048 (3)	0.1900 (3)	0.2443 (4)	0.0261 (7)	

### Atomic displacement parameters (Å^2^)

**Table:**

	*U*^11^	*U*^22^	*U*^33^	*U*^12^	*U*^13^	*U*^23^
Mg1	0.0125 (6)	0.0126 (6)	0.0301 (7)	0.0064 (5)	0.0002 (5)	0.0000 (5)
O1	0.0203 (14)	0.0149 (14)	0.0459 (19)	0.0108 (12)	0.0012 (13)	−0.0037 (13)
O2	0.0148 (13)	0.0184 (14)	0.0301 (16)	0.0063 (11)	0.0005 (11)	−0.0004 (11)
O3	0.0123 (13)	0.0136 (13)	0.0477 (19)	0.0041 (11)	0.0005 (12)	0.0017 (13)
O1S	0.0328 (18)	0.0305 (17)	0.0361 (18)	0.0169 (15)	−0.0057 (14)	0.0031 (14)
C2	0.0127 (17)	0.0148 (17)	0.029 (2)	0.0085 (14)	−0.0008 (14)	−0.0002 (14)
C3	0.0139 (17)	0.0114 (16)	0.030 (2)	0.0056 (14)	−0.0003 (14)	−0.0002 (14)
C4	0.0185 (19)	0.0194 (19)	0.027 (2)	0.0081 (16)	−0.0016 (15)	0.0009 (15)
C1	0.0179 (18)	0.0160 (17)	0.027 (2)	0.0112 (15)	0.0021 (15)	−0.0001 (15)
N1S	0.033 (2)	0.044 (3)	0.032 (2)	0.019 (2)	−0.0002 (17)	0.0045 (19)
C1S	0.028 (2)	0.033 (2)	0.039 (3)	0.016 (2)	0.003 (2)	0.006 (2)
C2S	0.039 (3)	0.067 (4)	0.034 (3)	0.018 (3)	−0.001 (2)	−0.001 (3)
C3S	0.064 (4)	0.054 (4)	0.044 (3)	0.039 (3)	−0.005 (3)	0.010 (3)
O4	0.0182 (14)	0.0281 (16)	0.0316 (17)	0.0112 (13)	0.0011 (12)	0.0005 (13)

### Geometric parameters (Å, °)

**Table:**

Mg1—O1	2.008 (3)	C3—H3	0.9300
Mg1—O2	2.132 (3)	C4—O4	1.238 (5)
Mg1—O2^i^	2.135 (3)	C4—H4A	0.9300
Mg1—O3	2.047 (3)	C1—O3^v^	1.248 (5)
Mg1—O4^ii^	2.080 (3)	N1S—C1S	1.314 (6)
Mg1—O1S	2.115 (4)	N1S—C3S	1.456 (8)
O1—C1	1.251 (5)	N1S—C2S	1.455 (7)
O2—C4	1.260 (5)	C1S—H1S	0.9300
O3—C1^i^	1.248 (5)	C2S—H2S1	0.9600
O1S—C1S	1.243 (6)	C2S—H2S2	0.9600
C2—C3	1.390 (5)	C2S—H2S3	0.9600
C2—C3^iii^	1.392 (5)	C3S—H3S1	0.9600
C2—C1	1.507 (5)	C3S—H3S2	0.9600
C3—C2^iv^	1.392 (5)	C3S—H3S3	0.9600
			
O1—Mg1—O3	89.26 (13)	C1S—O1S—Mg1	122.9 (3)
O1—Mg1—O4^ii^	170.66 (15)	C3—C2—C3^iii^	119.2 (4)
O3—Mg1—O4^ii^	93.03 (14)	C3—C2—C1	120.4 (4)
O1—Mg1—O1S	86.23 (15)	C3^iii^—C2—C1	120.3 (3)
O3—Mg1—O1S	90.84 (15)	C2—C3—C2^iv^	120.8 (4)
O4^ii^—Mg1—O1S	84.69 (14)	C2—C3—H3	119.6
O1—Mg1—O2	89.14 (13)	C2^iv^—C3—H3	119.6
O3—Mg1—O2	177.59 (15)	O4—C4—O2	127.0 (4)
O4^ii^—Mg1—O2	88.83 (13)	O4—C4—H4A	116.5
O1S—Mg1—O2	90.85 (14)	O2—C4—H4A	116.5
O1—Mg1—O2^i^	96.73 (14)	O3^v^—C1—O1	125.8 (4)
O3—Mg1—O2^i^	89.05 (13)	O3^v^—C1—C2	118.0 (3)
O4^ii^—Mg1—O2^i^	92.36 (13)	O1—C1—C2	116.2 (4)
O1S—Mg1—O2^i^	177.04 (15)	C1S—N1S—C3S	120.3 (5)
O2—Mg1—O2^i^	89.35 (15)	C1S—N1S—C2S	122.2 (5)
O1—Mg1—Mg1^i^	123.18 (13)	C3S—N1S—C2S	117.3 (5)
O3—Mg1—Mg1^i^	72.76 (10)	O1S—C1S—N1S	124.4 (5)
O4^ii^—Mg1—Mg1^i^	66.10 (9)	O1S—C1S—H1S	117.8
O1S—Mg1—Mg1^i^	144.98 (12)	N1S—C1S—H1S	117.8
O2—Mg1—Mg1^i^	106.68 (9)	N1S—C2S—H2S1	109.5
O2^i^—Mg1—Mg1^i^	32.42 (8)	N1S—C2S—H2S2	109.5
O1—Mg1—Mg1^v^	65.14 (11)	H2S1—C2S—H2S2	109.5
O3—Mg1—Mg1^v^	145.14 (13)	N1S—C2S—H2S3	109.5
O4^ii^—Mg1—Mg1^v^	116.15 (10)	H2S1—C2S—H2S3	109.5
O1S—Mg1—Mg1^v^	109.51 (11)	H2S2—C2S—H2S3	109.5
O2—Mg1—Mg1^v^	32.48 (8)	N1S—C3S—H3S1	109.5
O2^i^—Mg1—Mg1^v^	72.03 (9)	N1S—C3S—H3S2	109.5
Mg1^i^—Mg1—Mg1^v^	100.84 (5)	H3S1—C3S—H3S2	109.5
C1—O1—Mg1	137.5 (3)	N1S—C3S—H3S3	109.5
C4—O2—Mg1	120.5 (3)	H3S1—C3S—H3S3	109.5
C4—O2—Mg1^v^	124.4 (3)	H3S2—C3S—H3S3	109.5
Mg1—O2—Mg1^v^	115.10 (14)	C4—O4—Mg1^vi^	137.3 (3)
C1^i^—O3—Mg1	128.3 (3)		

Symmetry codes: (i) *x*−*y*, *x*−1, −*z*+1; (ii) −*y*+1, *x*−*y*−1, *z*; (iii) −*y*+1, *x*−*y*, *z*; (iv) −*x*+*y*+1, −*x*+1, *z*; (v) *y*+1, −*x*+*y*+1, −*z*+1; (vi) −*x*+*y*+2, −*x*+1, *z*.
